# Ca 125 is an independent prognostic marker in resected pancreatic cancer of the head of the pancreas

**DOI:** 10.1007/s13304-023-01587-4

**Published:** 2023-08-03

**Authors:** Niccolò Napoli, Emanuele F. Kauffmann, Michael Ginesini, Lucrezia Lami, Carlo Lombardo, Fabio Vistoli, Daniela Campani, Ugo Boggi

**Affiliations:** 1 Division of General and Transplant Surgery, University of Pisa, Pisa, Italy; 2 Division of Pathology, University of Pisa, Pisa, Italy

**Keywords:** Pancreatic ductal adenocarcinoma, Pancreatic cancer, Survival, Prognosis, CA 125, CA 19-9

## Abstract

The prognostic value of carbohydrate antigen 125 (Ca 125) is emerging also in pancreatic cancer (PDAC). In this study, we aim to define the prognostic value of Ca 125 in resected PDAC of the head of the pancreas. This is a single-center, retrospective study. Data from patients with a pre-operative assay of Ca 125 who underwent a pancreatic resection for PDAC between 2010 and 2018 were analyzed. As per National Comprehensive Cancer Guidelines, tumors were classified in resectable (R-PDAC), borderline resectable (BR-PDAC), and locally advanced (LA-PDAC). The Kaplan–Meier method was used to evaluate the overall survival. Cox proportional hazard regression was used to evaluate the role of pre-operative Ca 125 in predicting survival (while adjusting for confounders). The maximally selected log-rank statistic was used to identify a Ca 125 cut-off defining two groups with different survival probability. Inclusion criteria were met by 207 patients (R-PDAC: 80, BR-PDAC: 91, and LA-PDAC: 36). Ca 125 predicted overall survival before and after adjusting for confounding factors in all categories of anatomic resectability (R-PDAC: HR = 4.3; *p* = 0.0249) (BR-PDAC: HR = 7.82; *p* = 0.0024) (LA-PDAC: HR = 11.4; *p* = 0.0043). In BR-PDAC and LA-PDAC (*n* = 127), the division in two groups (high vs. low Ca 125) correlated with T stage (*p* = 0.0317), N stage (*p* = 0.0083), mean LN ratio (*p* = 0.0292), and tumor grading (*p* = 0.0143). This study confirmed the prognostic value of Ca125 in resected pancreatic cancer and, therefore, the importance of biologic over anatomic resectability. Ca 125 should be routinely assayed in surgical candidates with PDAC.

## Introduction

Pancreatic ductal adenocarcinoma (PDAC) is a dreadful disease marked by an extremely high case fatality rate [1]. A slight increase in survival has been recently noted, thanks to the availability of effective chemotherapy (e.g., FOLFIRINOX) [2], but radical tumor resection remains the main pillar of all treatment protocols aiming at cure. Actually, there is no competition between chemotherapy and surgery. Both treatments should be delivered to most patients with seemingly localized PDAC to increase the probability of cure [2]. Actually, pre-operative chemotherapy has increased the pool of surgical candidates [3] permitting surgery in previously unresectable patients, mostly by excluding those who develop distant metastasis while under treatment [4]. It has been suggested that in PDAC development of distant metastasis precedes pancreatic tumor formation [5]. This theory could appear too pessimistic, but autopsy studies demonstrated that 85%–90% of the patients die of recurrent disease after resection of PDAC, with 70%–85% being killed by distant metastasis [6, 7]. Despite the yet limited possibility to detect occult micro-metastasis at the time of diagnosis [8, 9], the high rate of failure from distant metastasis following resection of seemingly early PDAC [10] has prompted the origin of the concept of biological resectability, that is taking over the traditional view of anatomic resectability [11]. While molecular biology could eventually provide more robust evidence, the current concept of biological resectability is mostly based on pre-operative levels of carbohydrate antigen 19.9 (Ca 19.9) [12, 13]. However, Ca 19.9 has some basic flaws making it an imperfect tool for prognostic anticipation in some patients. Ca 19.9 can be falsely negative in approximately 5–10% of the general population, having non-sialylated Lewis blood group antigen [14]. These patients, defined as “non-secretors”, are those showing serum levels of Ca 19.9 ≤ 2 U/mL [15]. On the other hand, Ca 19.9 can be falsely positive in patients with obstructive jaundice [16] and/or cholangitis [17]. Some of these patients may have a PDAC, making them true positive, but jaundice and cholangitis interfere with the levels of Ca 19.9 and, hence, with the ensuing prognostic implications of this tumor marker. Limitations of Ca 19.9 have provided impetus to the search of other tumor markers for PDAC [15].

Circulating Ca 125 is the cleaved product of Mucin 16 (MUC 16), the largest membrane glycoprotein. Ca 125 is a well-known marker in ovarian cancer [18] predicting tumor progression and recurrence. Ca 125 is a promising marker for PDAC. In PDAC, Ca 125 enhances cell motility and tumor invasion capability, by binding mesothelin [19, 20], promotes the expression of receptor-interacting protein kinases 4, that regulates the proteasome-mediated phosphatidylethanolamine-binding protein 1 degradation, and activates the RAF1/MEK/ERK signaling [21]. Moreover, Ca 125 may facilitate binding of cancer cells to vascular endothelium during intravasation, and interacts with platelets eventually promoting the development of liver metastases [22]. Ca 125 can outperform Ca 19.9 in the prediction of micro-metastases [23], and several studies have shown that it predicts prognosis in PDAC [24–26]. High levels of MUC 16/Ca 125 neoantigens, promoting a MUC16-specific T cell immunity, were found in patients surviving long-term with a PDAC [27], and a monoclonal antibody (AR9.6) has been recently proposed as targeted therapy against MUC16 in PDAC [28]. It is, therefore, clear that Ca 125 could provide new prognostic information in PDAC.

The aim of this study was to provide further insights into the prognostic value of pre-operative Ca 125 based on anatomic criteria for pancreatic resection: resectable PDAC (R-PDAC), borderline resectable PDAC (BR-PDAC), and locally advanced PDAC (LA-PDAC).

## Methods

This is a single-center retrospective cohort study aiming to define the prognostic implications of Ca 125 in different categories of PDAC resected by means of pancreatoduodenectomy (PD) or total pancreatectomy (TP): R-PDAC, BR-PDAC, and LA-PDAC. Patients with left-sided PDAC were excluded due to different genetic characteristics and prognostic implications between PDAC of the head and of the body–tail of the pancreas [29]. The study was approved by the Institutional Ethical Board of the University of Pisa and was performed according to the principles of the Declaration of Helsinki [30] and the Strengthening the Reporting of Observational studies in Epidemiology (STROBE) guidelines on reporting on observational studies [31].

A prospectively maintained database was queried to identify patients who underwent curative pancreatic resection for PDAC between January 2010 and November 2018, at the Division of General and Transplant Surgery of Pisa University Hospital. For diagnostic and staging purposes, all patients had at least a high-quality thoraco-abdominal computed tomography (CT) scan performed within four weeks of surgery [32]. Additional diagnostic studies were performed as required. To be eligible for this study, patients were also required to have a pre-operative assay of both Ca 19.9 and Ca 125. Exclusion criteria were: distal pancreatectomy, post-operative mortality, and unavailability of detailed follow-up information. Follow-up information was updated on April 24, 2023.

Based on National Comprehensive Cancer Network (NCCN) guidelines [32], resected tumors were classified into R-PDAC, BR-PDAC, and LA-PDAC. Patients with R-PDAC were operated upfront, while those with BR-PDAC and LA-PDAC received pre-operative chemotherapy and/or chemoradiotherapy. Patients from all groups were considered operable when in good performance status, if there was no evidence of distant metastasis, and when Ca 19.9 levels were low or declined following pre-operative chemotherapy or chemoradiotherapy. Regarding vascular involvement, provided that the above criteria were meet, the only absolute contraindication to resection was inability to reconstruct the vessels. Our group has provided several publications on how to select patients with BR-PDAC and LA-PDAC for surgery, prognostic implications of vascular involvement, and technical details of pancreatectomy with resection and reconstruction of peripancreatic vessels [26, 33–35]. More recently, our group also described the “cold triangle” technique that we use for radical resection of PDAC in pancreatoduodenectomy [36]. Details of specimen pathology were also reported previously [37]. Margins were assessed circumferentially and were considered positive if cancer cells were found ≤ 1 mm of any margin [38]. TNM stage was defined according to the 8th edition of the American Joint Committee on Cancer staging manual [39]. Slides from all patients included in this study were revised by a dedicated pathologist (D.C.).

### Examined variables

Pre-operative Ca 19.9 and Ca 125 were available for all patients included in this study. For those who received pre-operative oncology treatments Ca 19.9 levels were also available before and during treatment. The cut-off values were 37 U/mL and 35 U/mL for Ca 19.9 and Ca 125, respectively. Patients with a Ca 19.9 ≤ 2 U/mL were considered “non-secretors” [15].

The following variables, possibly acting as prognostic confounders, were also evaluated: age, American Society of Anesthesiologists (ASA) score, length of hospital stay, incidence of severe post-operative complications (defined according to Clavien–Dindo and considered severe when > 3a) [40], and the comprehensive complication index [41]. Ca 19-9 and Ca 125 serum level, age, length of hospital stay, and comprehensive complication index were considered continuous variables.

Time-to-event endpoints were defined according to DATECAN (Definition for Assessment of Time-to-event Endpoints in CANcer trials) [42]. Namely, overall survival (OS) was defined as the time between the first treatment (first treatment-OS), either surgery or chemotherapy, and death. The follow-up time started from the day of the first delivered treatment. Considering the purpose of the study, an additional overall survival (surgery-OS) was calculated as the time between surgery and death.

### Statistical analysis

Categorical variables were summarized as frequencies, percentages, and rates. Continuous variables were expressed as mean ± SD, if normally distributed, or as median and interquartile range (IQR), if not. Normality distribution was checked by the Shapiro–Wilk test.

The Pearson Chi-square test and the Fisher’s Exact test (if group population < 5) were used to compare categorical variables between different groups. The Cochran Armitage test for trend and the Wilcoxon/Kruskal–Wallis test were preferred for comparing ordinal and continuous variables, respectively.

Time-to-event endpoints (OS) were estimated using the non-parametric Kaplan–Meier method. The log-rank test was used for comparing the survival between different groups.

The univariate Cox proportional hazard regression was used to check the prognostic relevance of serum biomarkers. The hazard ratio (HR) was used as an estimation of the effect size, defined as crude HR (Crude-HR). The other pre-operative and post-operative variables, resulting as possible confounders from the univariate Cox proportional hazard regression, were inserted in a multivariate Cox proportional hazard model to estimate an adjusted HR (Adjusted-HR) for serum biomarkers. The proportional hazards test (cox.zph R function) and the Schoenfeld/Martingale residuals plots were used to test the Cox proportional hazard assumptions. Non-linearity tests (Martingale versus covariate, Gam-Poisson, smoothing splines), influential observations, and outliers for continuous covariates were also checked.

The maximally selected log-rank statistic (*maxstat* R package) was used to identify the optimal cut-off point of biomarker level for predicting the long-term survival. This method allowed to identify a value that reflect the most significant (the smallest *p*-value at the log-rank test) discrepancy in term of survival between two different groups: low vs. high values of the serum marker.

For the statistical significance of the test, a power = 80%, *p* < 0.05, two-tailed significance level were used.

All statistical analyses were carried out with JMP® Pro 16.0.0 software package for Mac (Copyright© SAS Institute Inc., SAS campus Drive, Cary, NC, USA) and R Package, R Core Team (2014): A language and Environment for Statistical Computing (R Foundation for Statistical Computing, Vienna AT) version 4.3.0(2023-04-21) using the Rstudio by Posit, the BlueSky Statistics software 10.3.1 platform (BlueSky Statistics LLC, Chicago, IL, USA), the Jamovi (version 2.3) computer software (*jamovi* project—2022, retrieved from https://www.jamovi.org) as IDEs for R, as well as *survminer* and *maxstat* R packages.

## Results

**Fig. 1:**
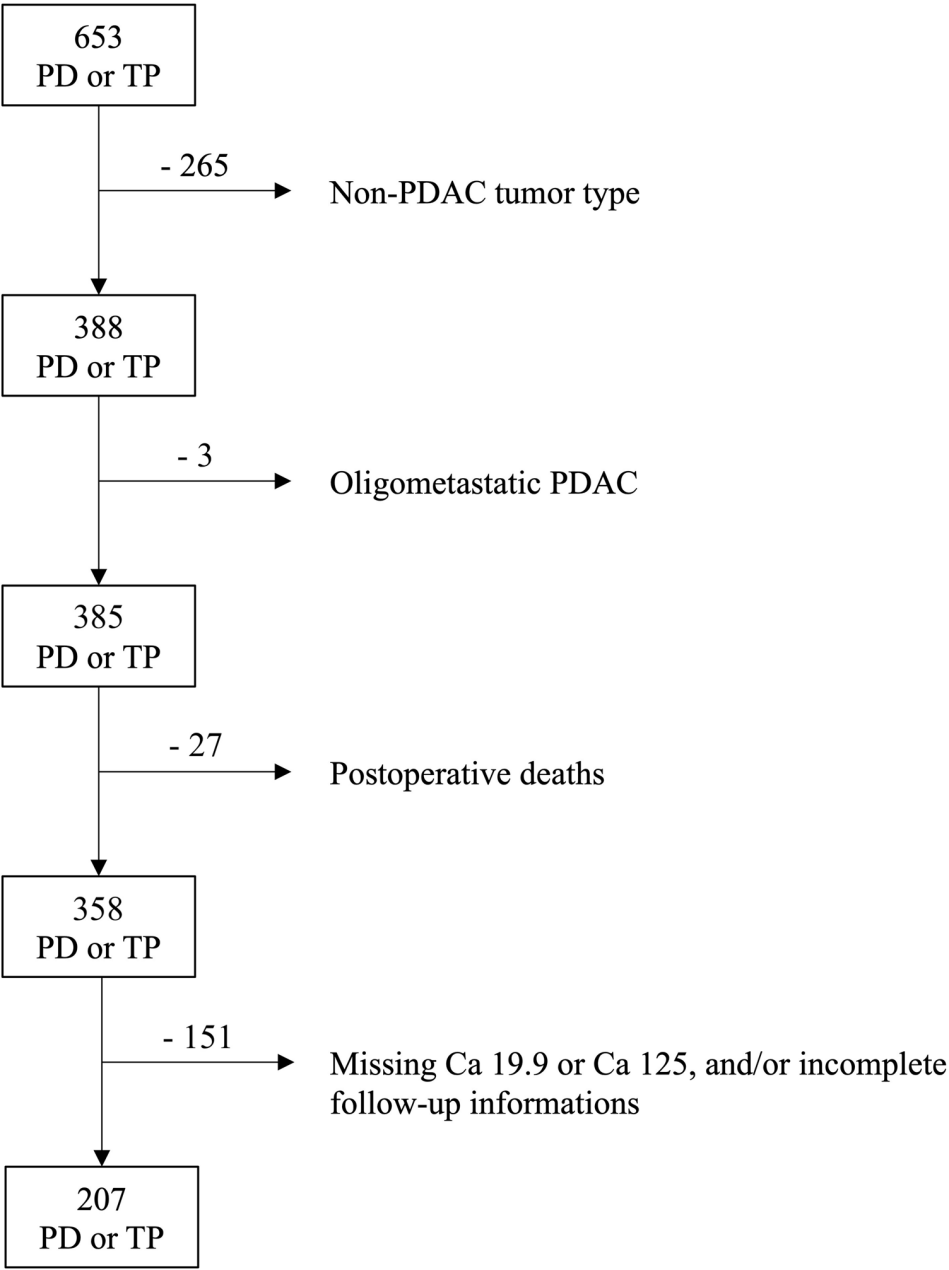
Flow chart based on exclusion criteria

During the study period, 653 patients underwent PD or TP. Following application of inclusion and exclusion criteria, 207 patients were available for the purpose of this study (Fig. 1). There were 80 R-PDAC (38.6%), 91 BR-PDAC (43.9%), and 36 LA-PDAC (17.4%). Baseline characteristics, details of operative procedures, post-operative results, pathological findings, and follow-up information of these patients are presented in Table 1.

**Table 1:** Baseline characteristics, details of operative procedures, post-operative results, pathological findings, and follow-up of patients with resectable (R-PDAC), borderline resectable (BR-PDAC), and locally advanced (LA-PDAC) PDAC

	Overall population	R-PDAC	BR-PDAC	LA-PDAC	*p*
Number	207	80	91	36	
*Pre-operative characteristics*					
Male gender	100 (48.3%)	36 (45%)	46 (50.6%)	18 (50%)	0.750
Median age (years)	67 (60–74)	71 (62–74)	70 (62–77)	60 (55–65.5)	** < 0.0001**
Arterial hypertension	93 (44.9%)	39 (48.8%)	41 (45.1%)	13 (36.1%)	0.448
Heart disease	33 (15.9%)	17 (21.3%)	13 (14.3%)	3 (8.3%)	0.202
Chronic obstructive pulmonary disease	10 (4.8%)	5 (6.3%)	5 (5.5%)	0 (0%)	0.392
Diabetes	8 (3.9%)	20 (25%)	23 (25.3%)	9 (25%)	0.999
ASA^ score					
ASA I	8 (3.9%)	2 (2.5%)	3 (3.3%)	3 (8.3%)	0.310
ASA II	88 (42.5%)	25 (31.3%)	43 (47.3%)	20 (55.6%)	**0.0236**
ASA III	99 (47.8%)	43 (53.8%)	44 (48.4%)	12 (33.3%)	0.125
ASA IV	12 (5.8%)	10 (12.5%)	1 (1.1%)	1 (2.8%)	**0.0050**
Median ASA score	3 (2–3)	3 (2–3)	2 (2–3)	2 (2–3)	**0.0012**
Obstructive jaundice	106 (51.2%)	54 (67.5%)	48 (52.8%)	4 (11.1%)	** < 0.0001**
Weight loss	42 (20.3%)	9 (11.3%)	24 (26.4%)	9 (25%)	**0.0316**
Pre-operative chemotherapy	43 (20.8%)	0	12 (13.2%)	31 (86.1%)	** < 0.0001**
FOLFIRINOX^&^	32 (15.5%)	0	11 (12.1%)	21 (58.3%)	** < 0.0001**
Gemcitabine + Nab-paclitaxel^&^	5 (2.4%)	0	1 (1.1%)	4 (11.1%)	**0.0035**
Gemcitabine derivatives^&^	8 (18.6%)	0	0	8 (25.8%)	0.0821
N° cycles	8 (6–9)	NA	8 (5.3–9.8)	8 (6–9)	0.956
Ca 19.9 in non-secretors, *n*, (%)	16 (7.7%)	7 (8.8%)	8 (8.8%)	1 (2.8%)	0.577
Ca 19.9 in all secretors, median, U/mL	96.7 (20.3–355.3)	104.6 (42.9–439.8)	145.6 (15.6–477)	28 (16.8–92.4)	0.0540
Ca 19.9 in secretors receiving pre-operative oncology treatments, median, U/mL					
Before pre-operative oncology treatments	242.5 (73.9–559.8)	–	245 (1.6–1698.3)	240 (87.3–563.9)	0.662
Peak level	132 (33.9–477)	–	145.6 (15.6–477)	165 (66.4–567.9)	0.355
Drop percentage, %	74 (39–93)	–	18.3 (− 311.4 to 66)	76.6 (49.7–93)	0.0529
Ca 125, median, U/mL	15.3 (10.1–26.0)	16.5 (10.8–27.4)	16.3 (10.6–28.9)	10.9 (7.7–18)	**0.00089**
*Details of operative procedures*					
Procedure					
Pancreatoduodenectomy	143 (69.1%)	75 (93.8%)	68 (74.7%)	0	** < 0.0001**
Total pancreatectomy	64 (30.9%)	5 (6.2%)	23 (25.3%)	36 (100%)	
Operative approach					
Open	168 (81.2%)	54 (67.5%)	78 (85.7%)	36 (100%)	** < 0.0001**
Robotic	39 (18.8%)	26 (32.5%)	13 (14.3%)	0	
Associated vascular procedures					
Vascular resection	135 (65.2%)	8 (10%)	91 (100%)	36 (100%)	** < 0.0001**
Isolated venous resection	96 (46.4%)	7 (8.8%)	89 (97.8%)	0	** < 0.0001**
Isolated arterial resection	1 (0.5%)	1 (1.3%)	0	0	0.560
Combined arterial and venous resection	38 (18.4%)	0	2 (2.2%)	36 (100%)	** < 0.0001**
Hepatic artery/ celiac trunk resection	25 (12.1%)	0	2 (2.2%)	23 (63.9%)	** < 0.0001**
Superior mesenteric artery resection	23 (11.1%)	1 (1.3%)	0	22 (61.1%)	** < 0.0001**
*Post-operative results*					
Length of hospital stay, median, days	18 (14–26)	16.5 (13–24)	19 (14–26)	20 (15–26.8)	0.0544
Post-operative complications (90 days)					
Clavien–Dindo grade 0	45 (21.7%)	13 (16.3%)	21 (23.1%)	11 (30.6%)	0.206
Clavien–Dindo grade 1	26 (12.6%)	12 (15%)	10 (11%)	4 (11.1%)	0.757
Clavien–Dindo grade 2	98 (47.3%)	45 (56.3%)	40 (44%)	13 (36.1%)	0.0913
Clavien–Dindo grade 3a	20 (9.7%)	8 (10%)	8 (8.8%)	4 (11.1%)	0.863
Clavien–Dindo grade 3b	11 (5.3%)	1 (1.3%)	10 (11%)	0	**0.0071**
Clavien–Dindo grade 4a	5 (2.4%)	1 (1.3%)	1 (1.1%)	3 (8.3%)	0.0784
Clavien–Dindo grade 4b	2 (1%)	0 (0%)	1 (1.1%)	1 (2.8%)	0.467
Clavien–Dindo > 3a	18 (8.7%)	2 (2.5%)	12 (13.2%)	4 (11.1%)	**0.0266**
Median Comprehensive Complication Index	20.9 (8.7–30.8)	20.9 (8.7–29.6)	22.6 (8.7–30.8)	20.9 (0–35.8)	0.868
*Pathology*					
T stage					
T1a	2 (1%)	0	1 (1.1%)	1 (2.8%)	0.467
T1b	0	0	0	0	–
T1c	33 (15.9%)	23 (28.8%)	9 (9.9%)	1 (2.8%)	**0.0002**
T2	140 (6.7%)	55 (68.8%)	65 (71.4%)	20 (55.6%)	0.218
T3	21 (10.1%)	2 (2.5%)	15 (16.5%)	4 (11.1%)	**0.0042**
T4	11 (5.3%)	0	1 (1.1%)	10 (27.8%)	** < 0.0001**
> T2	32 (15.5%)	2 (2.5%)	16 (17.6%)	14 (38.9%)	** < 0.0001**
N stage					
N0	29 (14%)	10 (12.5%)	16 (17.6%)	3 (8.3%)	0.384
N1	79 (38.2%)	30 (37.5%)	30 (33%)	19 (52.8%)	0.116
N2	99 (47.8%)	40 (50%)	45 (49.5%)	14 (38.9%)	0.497
TNM stage					
1A	14 (6.7%)	6 (7.5%)	7 (7.7%)	1 (2.8%)	0.666
1B	12 (5.8%)	5 (6.3%)	5 (5.5%)	2 (5.6%)	1.000
2A	4 (1.9%)	0	4 (4.4%)	0	0.136
2B	72 (34.8%)	29 (36.3%)	30 (33%)	13 (36.1%)	0.889
3	105 (50.7%)	40 (50%)	45 (49.5%)	20 (55.6%)	0.814
Grading					
1	0	0	0	0	
2	160 (77.3%)	59 (73.8%)	74 (81.3%)	27 (75%)	0.468
3	47 (22.7%)	21 (26.3%)	17 (18.7%)	9 (25%)	0.468
R1	77 (37.2%)	35 (43.8%)	33 (36.3%)	9 (25.0%)	0.150
Examined lymph nodes, median, number	52 (41–72)	44.5 (37–54.3)	55 (41–74)	77 (65.5–101)	** < 0.0001**
Metastatic lymph nodes, median, number	3 (1–7)	4 (1.3–7)	4 (1–7)	3 (2–6.8)	0.952
Lymph node ratio, median	0.07 (0.02–0.14)	0.07 (0.03–0.17)	0.07 (0.02–0.15)	0.03 (0.02–0.07)	**0.0195**
LODDS^&^, median	− 1.1 (− 1.5 to − 0.76)	− 1.02 (− 1.45 to − 0.65)	− 1.08 (− 1.54 to − 0.74)	− 1.40 (− 1.59 to 1.08)	**0.0072**
Perineural invasion	177 (85.5%)	70 (87.5%)	78 (85.7%)	29 (80.6%)	0.615
Vein infiltration	92 (44.4%)	4 (5.0%)	64 (70.3%)	24 (66.7%)	** < 0.0001**
Artery infiltration	13 (6.3%)	0	2 (2.2%)	10 (27.8%)	** < 0.0001**
Long-term follow-up*					
Median duration of follow-up	21.6 (12.4–51.8)	26.9 (12.7–56.4)	18.9 (11.2–41.2)	19.6 (14–46.2)	
First treatment-OS					
12-month mortality	39 (19.6%)	15 (19.5%)	22 (25.3%)	2 (5.7%)	**0.0392**
36-month mortality	113 (63.5%)	36 (53.7%)	56 (70.9%)	21 (65.6%)	0.0964
60-month mortality	132 (77.2%)	45 (72.6%)	62 (80.5%)	25 (78.1%)	0.536
Median duration of follow-up	18.5 (10.9–51.2)	27.4 (12.6–56.4)	16.8 (10–33.8)	15.8 (8–46.3)	
Surgery-OS					
12-month mortality	52 (26.3%)	15 (19.5%)	24 (27.6%)	13 (38.2%)	0.110
36-month mortality	117 (65.7%)	36 (53.7%)	58 (73.4%)	23 (71.9%)	**0.0319**
60-month mortality	132 (77.6%)	45 (72.6%)	62 (81.6%)	25 (78.1%)	0.450

*p* < 0.05 are in bold

^*^ Patients alive before the respective time points are censored; ^&^some patients underwent more neoadjuvant protocols in combination; ^ASA = American Society of Anesthesiologists physical status classification; ^&^LODDS = log odds of positive lymph nodes

**Fig. 2:**
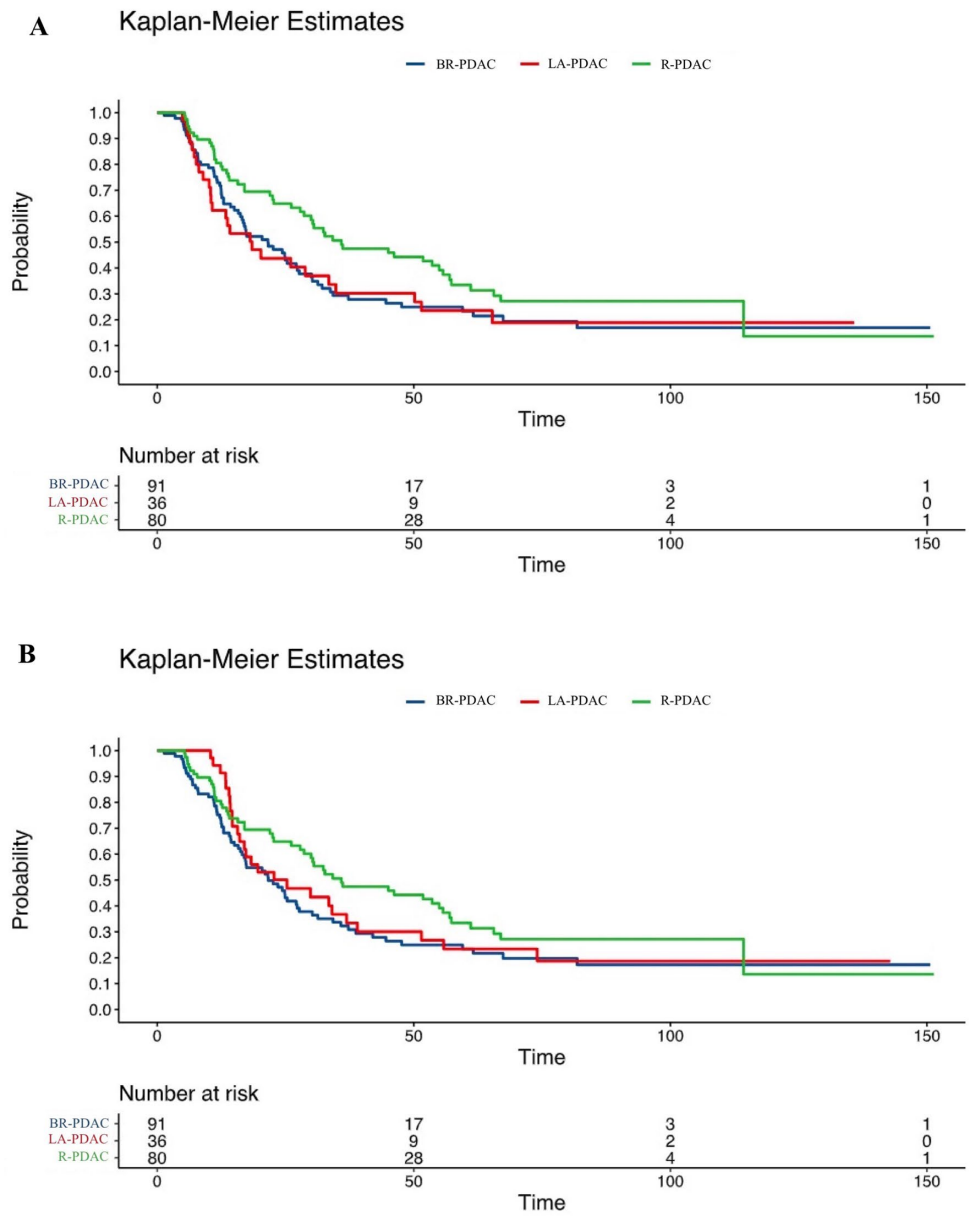
**A** Kaplan–Meier curve for R-PDAC, BR-PDAC, and LA-PDAC regarding surgery-OS (*p* = 0.0718); **B** Kaplan–Meier curve for R-PDAC, BR-PDAC, and LA-PDAC regarding first treatment-OS (*p* = 0.141)

The overall rate of Ca 19.9 “non-secretors” was 8%. The 191 Ca 19.9 secretors had a median Ca 19.9 level of 132.0 U/mL (33.9–477). Ca 19.9 levels did not predict category in R-PDAC, possibly due to the higher use of neoadjuvant chemotherapy in the other study groups resulting in lower levels of Ca 19.9. LA-PDAC was more frequently in stage > T2 (*p* < 0.0001), and showed a higher median number of examined lymph nodes (*p* < 0.0001), a lower lymph node ratio (*p* = 0.0195), a lower log odds of positive lymph nodes (*p* = 0.0072), and a higher proportion of patients with vein and artery invasion (for both *p* =  < 0.0001). With a median level of 15.3 U/mL (10.1–26.0), Ca 125 strongly predicted category of anatomic resectability (*p* = 0.00089). Survival curves based on category of anatomic resectability are presented in Fig. 2. Median first treatment-OS was 27.3 (13.4–65.6) months in the overall population and 35.9 (14–114.3), 21.6 (12.2–47.6), and 25.3 (14.6–55.8) months in PR-PDAC, BR-PDAC and LA-PDAC (*p* = 0.141), respectively. Median surgery-OS was 26.2 (11.6–65.3) months in the overall population and 35.9 (14–114.3), 21.6 (11.6–47.6) and 18.5 (8.9–51.5) months in PR-PDAC, BR-PDAC, and LA-PDAC (*p* = 0.0718), respectively.

### Identification of prognostic factors for overall survival

Factors predicting either first treatment-OS or surgery-OS were identified by univariate Cox proportional hazard regression analysis (Table 2).

**Table 2:** Identification of factors influencing OS by Cox proportional hazard regression analysis

	First treatment-OS						Surgery-OS					
	R-PDAC		BR-PDAC		LA-PDAC		R-PDAC		BR-PDAC		LA-PDAC	
	RR (IQR)	*p*	RR (IQR)	*p*	RR (IQR)	*p*	RR (IQR)	*p*	RR (IQR)	*p*	RR (IQR)	*p*
Median age (years)^	1.006 (0.98–1.04)	0.708	1.03 (1.004–1.06)	**0.0234**	1.05 (0.99–1.11)	0.0672	1.006 (0.98–1.04)	0.708	1.03 (1.002–1.06)	**0.0389**	1.04 (0.99–1.09)	0.137
	1.28 (0.36–4.68)		3.58 (1.20–10.90)		3.96 (0.86–16.71)		1.28 (0.36–4.68)		3.23 (1.07–9.92)		2.93 (0.67–11.63)	
Male gender	1.34 (0.76–2.36)	0.320	0.76 (0.47–1.24)	0.274	1.07 (10.49–2.32)	0.873	1.34 (0.76–2.36)	0.320	0.80 (0.49–1.30)	0.364	0.96 (0.44–2.09)	0.918
ASA* score > 2	1.12 (0.62–2.03)	0.706	1.23 (0.75–2.01)	0.406	0.99 (0.44–2.24)	0.992	1.12 (0.62–2.03)	0.706	1.21 (0.74–1.97)	0.455	1.02 (0.45–2.30)	0.957
Median LOH (days)^	1.01 (0.99–1.03)	0.208	1.007 (0.99–1.02)	0.395	0.99 (0.96–1.02)	0.799	1.01 (0.99–1.03)	0.208	1.007 (0.99–1.02)	0.386	1.002 (0.97–1.02)	0.896
	3.29 (0.44–18.35)		1.69 (0.44–5.12)		0.74 (0.05–5.52)		3.29 (0.44–18.35)		1.70 (0.45–5.11)		1.17 (0.07–9.15)	
Clavien > 2	0.97 (0.41–2.31)	0.953	0.94 (0.53–1.66)	0.831	0.96 (0.38–2.41)	0.925	0.97 (0.41–2.31)	0.953	0.97 (0.55–1.71)	0.913	1.02 (0.40–2.56)	0.970
Clavien > 3a	6.41 (0.83–49.65)	0.080	1.23 (0.64–2.36)	0.533	5.00 (1.43–17.55)	**0.0119**	6.41 (0.83–49.65)	0.08	1.30 (0.68–2.50)	0.424	4.62 (1.46–14.64)	**0.0092**
Median CCI^	0.99 (0.97–1.01)	0.730	1.00 (0.98–1.02)	0.631	1.00 (0.98–1.03)	0.663	0.99 (0.97–1.01)	0.730	1.00 (0.99–1.02)	0.612	1.01 (0.99–1.03)	0.423
	0.82 (0.27–2.53)		1.25 (0.50–3.01)		1.35 (0.35–5.11)		0.82 (0.27–2.53)		1.26 (0.51–3.04)		1.71 (0.45–6.32)	

*p* < 0.05 are in bold

^ Continuous factors: RR for unit in the first line, RR for range in second line; *ASA = American Society of Anesthesiologists physical status classification

In BR-PDAC, age (continuous value) was the only factor predicting survival (HR for unit = 1.03, HR for range = 3.58, *p* = 0.0234 for first treatment-OS; HR for unit = 1.03, HR for range = 3.23, *p* = 0.0389 for surgery-OS). In LA-PDAC severe post-operative complications (Clavien–Dindo > 3a) were the only prognostic factor (HR = 5.00, *p* = 0.0119 for first treatment-OS; HR = 4.62, *p* = 0.0092 for surgery-OS).

### Prognostic value of Ca 19.9 and Ca 125 in R-PDAC

Fifty-four (67.5%) patients had high pre-operative bilirubin levels and 7 (8.8%) were Ca 19.9 “non-secretors”. No patient in this group received neoadjuvant chemotherapy. Unplanned vein resection was required in 7 patients (8.8%). One patient required unplanned arterial resection (1.3%).

**Fig. 3:**
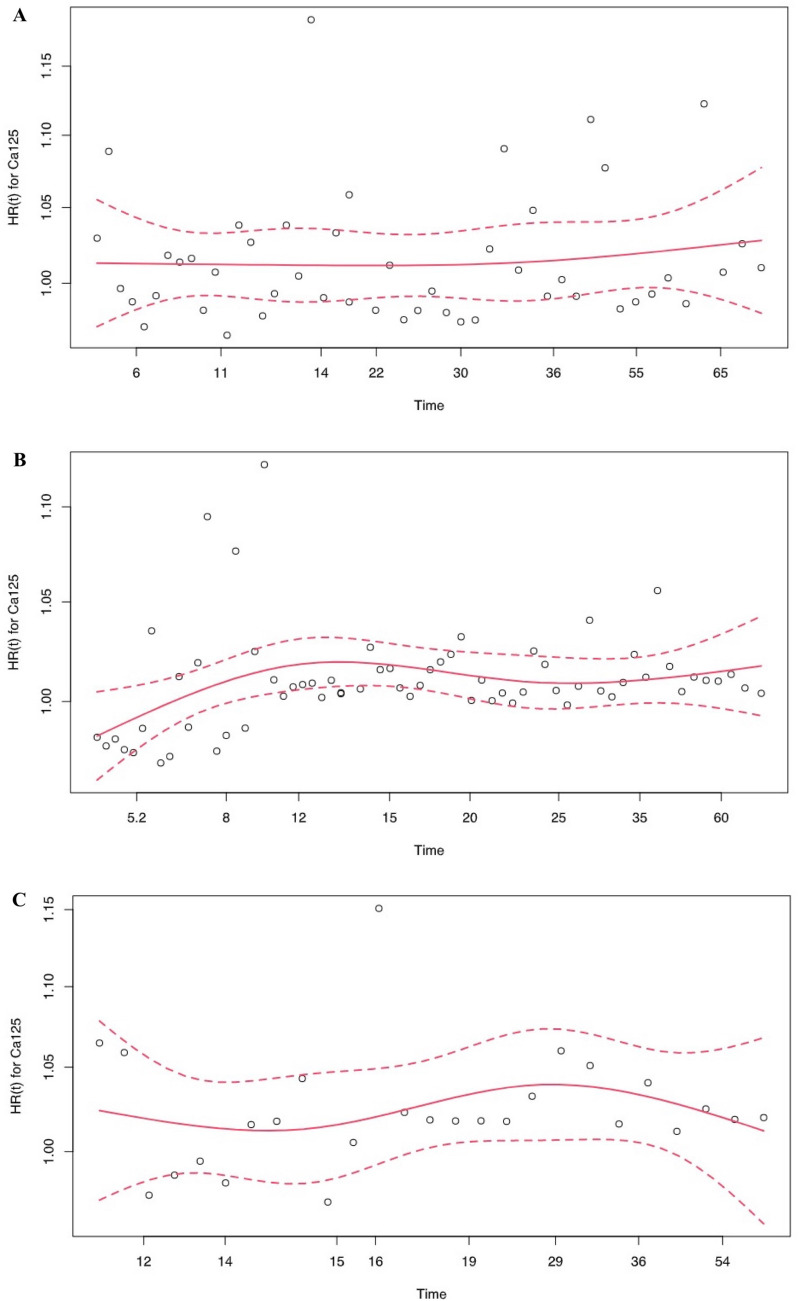
**A** Cox proportional hazard regression evaluating the ability of Ca 125 in the prediction of OS in R-PDAC (*p* = 0.0249); **B** Cox proportional hazard regression evaluating the ability of Ca 125 in the prediction of first treatment-OS (adjusted for age) in BR-PDAC (*p* = 0.0024); **C** Cox proportional hazard regression for evaluating CA 125 evaluating the ability of Ca 125 in the prediction of first treatment-OS (adjusted for development of severe post-operative complications) in LA-PDAC (*p* = 0.0043)

Pre-operative levels of Ca 19.9 predicted OS (HR for unit = 1.0003, HR for range = 15.2, *p* = 0.0026; Hazard test *p* = 0.196). Pre-operative levels of Ca 125 also predicted OS (HR for unit = 1.01, HR for range = 4.3, *p* = 0.0249; Hazard test *p* = 0.582) (Fig. 3A).

### Prognostic value of Ca 19.9 and Ca 125 in BR-PDAC

Forty-eight (52.8%) patients had high pre-operative bilirubin levels and 8 (8.8%) were Ca 19.9 “non-secretors”. Twelve (13.2%) patients received neoadjuvant chemotherapy (FOLFIRINOX *n* = 11, Gemcitabine + Nab-Paclitaxel *n* = 1; median number of cycles = 8, IQR = 5.3–9.8). Vein resection was required in 89 patients (8.8%), including two patients (2.2%) who required combined arterial resection (in both patients a replaced right hepatic artery from the superior mesenteric artery).

Median Ca 19.9 was higher in patients with elevated bilirubin levels (236.7 U/mL; 36.3–672 U/mL) versus patients with normal bilirubin levels (95.7 U/mL; 11.1–303.7 U/mL) (*p* = 0.0414). Both before and after adjustment for age, pre-operative Ca 19.9 did not predict first treatment-OS (Crude *p* = 0.174, Adjusted *p* = 0.258) as well as surgery-OS (Crude *p* = 0.229, Adjusted *p* = 0.258). Also, peak of Ca 19.9 did not predict survival (first treatment-OS: Crude *p* = 0.141, Adjusted *p* = 0.261) (surgery-OS: Crude *p* = 0.163, Adjusted *p* = 0.289).

Pre-operative Ca 125 predicted survival before and after adjustment for age (unadjusted first treatment-OS: HR for unit = 1.01, HR for range = 9.61, *p* = 0.0007; Hazard test *p* = 0.0806) (adjusted first treatment-OS: HR for unit = 1.01, HR for range = 7.82, *p* = 0.0024; Hazard test *p* = 0.0645) (unadjusted first surgery-OS: HR for unit = 1.01, HR for range = 7.43, *p* = 0.0025; Hazard test *p* = 0.0727) (unadjusted first surgery-OS: HR for unit = 1.01, HR for range = 6.2, *p* = 0.0067; Hazard test *p* = 0.0566) (Fig. 3B).

### Prognostic value of Ca 19.9 and Ca 125 in LA-PDAC

Four (11.1%) patients had high pre-operative bilirubin levels. In these patients, the diagnosis of LA-PDAC was made during surgery, once the point of no returned had already been trespassed. These patients did not receive pre-operative chemotherapy. One patient was “non-secretor” (2.8%). Thirty-one (86.1%) patients received primary chemotherapy (FOLFIRINOX *n* = 21, Gemcitabine + Nab-Paclitaxel *n* = 4, other Gemcitabine combination *n* = 8) and were considered for resection after multidisciplinary discussion. All patients required vein resection. In addition, the celiac trunk and the superior mesenteric artery were resected and reconstructed in 23 and 22 patients, respectively.

Median Ca 19.9 decreased by 77%, from 240 U/mL (87.3–563.9 U/mL) to 28 U/mL (16.8–92.4 U/mL) (*p* = 0.0050) following neoadjuvant therapy. Both before and after adjustment for severe post-operative complications, pre-operative Ca 19.9 did not predict first treatment-OS (Crude *p* = 0.181, Adjusted *p* = 0.218) as well as surgery-OS (Crude *p* = 0.155, Adjusted *p* = 0.128). Also peak of Ca 19.9 did not predict survival (first treatment-OS: Crude *p* = 0.382, Adjusted *p* = 0.490) (surgery-OS: Crude *p* = 0.382, Adjusted *p* = 0.490). Median percentage of Ca 19.9 drop only predicted adjusted surgery-OS (HR for unit = 0.41, HR for range = 0.12, *p* = 0.0445; Hazard test *p* = 0.919).

Pre-operative Ca 125 predicted survival before and after adjustment for severe post-operative complications (unadjusted first treatment-OS: HR for unit = 1.02, HR for range = 8.3, *p* = 0.007; Hazard test *p* = 0.5701) (adjusted first treatment-OS: HR for unit = 1.02, HR for range = 11.4, *p* = 0.0043; Hazard test *p* = 0.676) (unadjusted surgery-OS: HR for unit = 1.03, HR for range = 13.9, *p* = 0.0042; Hazard test *p* = 0.0804) (unadjusted surgery-OS: HR for unit = 1.03, HR for range = 20.3, *p* = 0.002; Hazard test *p* = 0.1014) (Fig. 3C).

### Ca 125 cut-off level predicting survival

Cut-off levels of Ca 125 predicting survival were 16.9 U/mL, 19.3 U/mL and 13.2 U/mL for R-PDAC, BR-PDAC and LA-PDAC, respectively. The high-Ca 125 group accounted for 37 (46.3%), 34 (37.4%), and 13 (36.1%) patients in each study group, respectively.

**Fig. 4:**
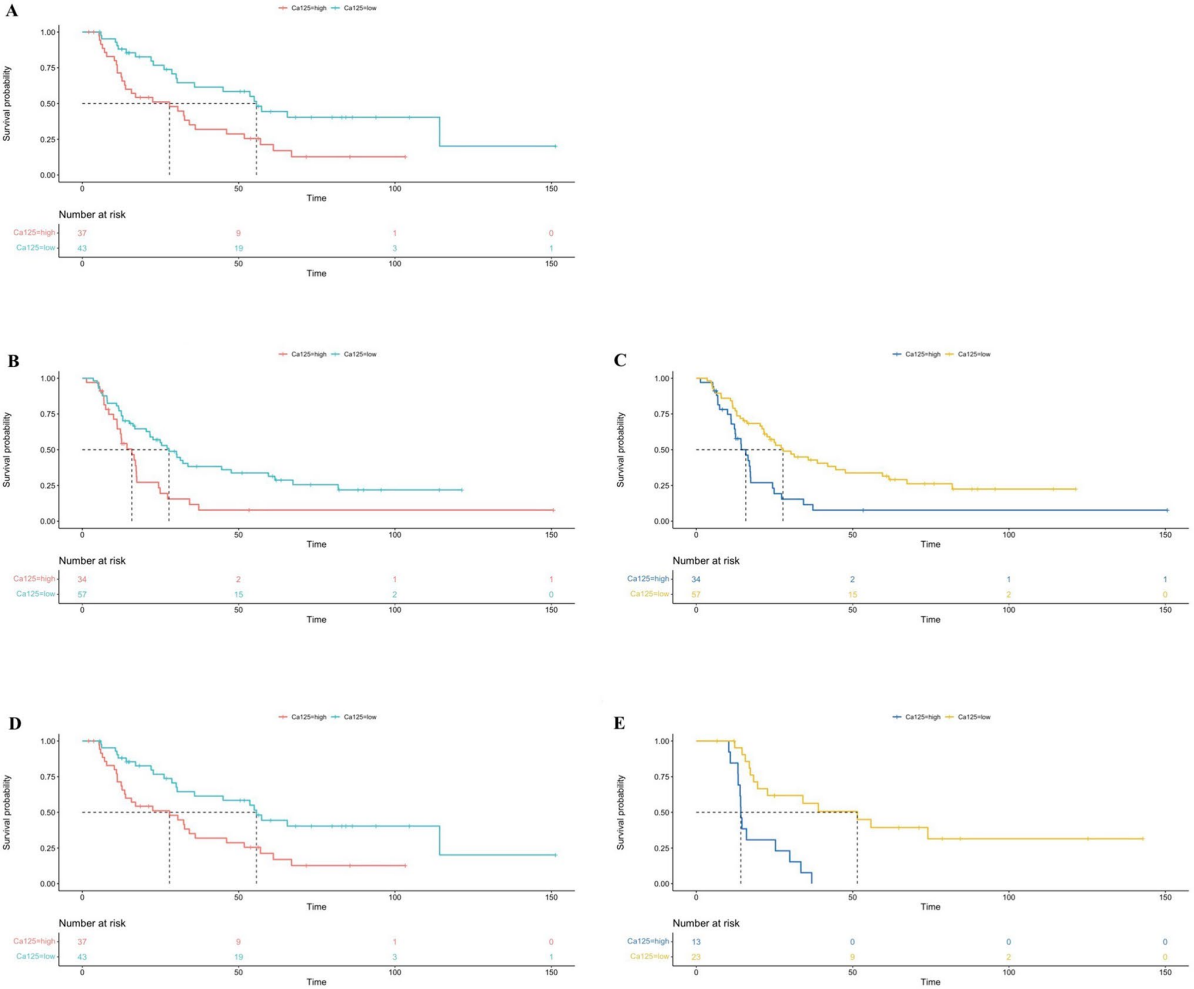
**A** Kaplan–Meier curve for high Ca 125 vs. low Ca 125 in the prediction of OS in R-PDAC (*p* = 0.004); **B** Kaplan–Meier curve for high Ca 125 vs. low Ca 125 in the prediction of surgery-OS in BR-PDAC (*p* = 0.004); **C** Kaplan–Meier curve for high Ca 125 vs. low Ca 125 in the prediction of first treatment-OS in BR-PDAC (*p* = 0.0016); **D** Kaplan–Meier curve for high-Ca 125 vs. low-Ca 125 groups in the prediction of surgery-OS in LA-PDAC (*p* = 0.0013); **E** Kaplan–Meier curve for high Ca 125 vs. low Ca 125 in the prediction of first treatment-OS in LA-PDAC (*p* < 0.0001)

In R-PDAC, median Ca 125 was 11.1 U/mL (8.3–13.3) in the low-risk group, and 29.3 U/mL (21.4–41.4) in the high-risk group (*p* < 0.0001). In BR-PDAC, median Ca 125 was 11.9 U/mL (9.7–15.8) in the low-risk group, and 38 U/mL (26.5–60.3) in the high-risk group (*p* < 0.0001). In LA-PDAC, median Ca 125 was 9.3 (6.5–9.8) U/mL in the low-risk group, and 21.1 (16.3–44.3) U/mL in the high-risk group (*p* < 0.0001). Low- and high-Ca 125 risk groups predicted survival in all categories of anatomic resectability [(R-PDAC, median OS: 55.7 [26.1–114.3] versus 27.9 [11.2–57] months; *p* = 0.004), (BR-PDAC, median first treatment-OS: 27.7 [12.9–82.8] versus 15.8 [10–24.4] months; *p* = 0.0016), (BR-PDAC, median surgery-OS: 27.7 (12.5–81.8) versus 15.8 (8.5–24.4) months; *p* = 0.004), (LA-PDAC, median first treatment-OS: 51.5 [18.3–NA] versus 14.2 [13.4–25.3] months; *p* < 0.0001), (LA-PDAC, median surgery-OS: 50.2 [10.4–NA] and 10.7 [6.8–18.5] months; *p* = 0.0013),] (Fig. 4A–E).

### Correlation between Ca.125 risk groups and PDAC pathology

Ca 125 risk groups correlated with pathology features potentially anticipating survival in a combined group made of BR-PDAC and LA-PDAC (*n* = 127).

The Ca 125 low-risk group included 80 patients (63%) and the Ca 125 high-risk group 47 patients (37%). Patients in the high-risk group had higher prevalence of > T2 PDAC (*p* = 0.0317), higher prevalence of N2 PDAC (*p* = 0.0083), higher lymph node ratio (*p* = 0.0292), and higher prevalence of G3 PDAC (*p* = 0.0143) (Table 3).

**Table 3:** Correlation between pathological features and different CA 125 expression group in the setting of BR-PDAC and LA-PDAC

	Low-CA125 (*n* = 80)	High-CA125 (*n* = 47)	OR (95% CI) / T (95% CI)^	*p*
*T*				
T1a	2 (2.5%)	0 (0%)	NA	0.530
T1b	0 (0%)	0 (0%)	NA	NA
T1c	9 (11.3%)	1 (2.1%)	0.17 (0.02–1.40)	0.090
T2	55 (68.8%)	30 (63.8%)	0.80 (0.38–1.72)	0.569
T3	7 (8.8%)	12 (25.5%)	3.58 (1.30–9.87)	**0.0105**
T4	7 (8.8%)	4 (8.5%)	0.97 (0.27–3.51)	1.000
T2–T3–T4	69 (86.3%)	46 (97.9%)	7.33 (0.92–58.75)	**0.0317**
T3–T4	14 (17.5%)	16 (34%)	2.43 (1.06–5.61)	**0.0341**
*N*				
N0	13 (16.3%)	6 (12.8%)	0.75 (0.27–2.14)	0.800
N1	37 (46.3%)	12 (25.5%)	0.40 (0.18–0.88)	**0.0206**
N2	30 (37.5%)	29 (61.7%)	2.69 (1.28–5.64)	**0.0083**
Median number of harvested nodes (IQR)	63 (44–80.8)	65 (49–83)	0.04 (− 9.28 to 9.67)	0.968
Median number metastatic nodes (IQR)	3 (1–6)	5 (2–10)	1.73 (− 0.27–4.00)	0.0865
Median LN ratio (IQR)	0.04 (0.02–0.11)	0.07 (0.04–0.15)	2.21 (0.004–0.07)	**0.0292**
Median LODDS^&^ (IQR)	− 1.26 (− 1.60 to − 0.86)	− 1.06 (− 1.36 to − 0.73)	1.96 (− 0.001 to 0.37)	0.0517
*Stage*				
1A	8 (10%)	0 (0%)	NA	0.0256
1B	4 (5%)	3 (6.4%)	1.30 (0.28–6.06)	0.709
2A	1 (1.3%)	3 (6.4%)	5.39 (0.54–53.35)	0.143
2B	32 (40%)	11 (23.4%)	0.46 (0.20–1.30)	0.0564
3	35 (43.8%)	30 (63.8%)	2.26 (1.08–4.76)	**0.0289**
2B + 3	67 (83.8%)	41 (87.2%)	1.33 (0.47–3.76)	0.595
*Grading*				
2	69 (86.3%)	32 (68.1%)	0.34 (0.14–0.82)	**0.0143**
3	11 (13.8%)	15 (31.9%)		
*R*				
R1	28 (35%)	14 (29.8%)	0.79 (0.36–1.71)	0.547
Perineural invasion	67 (83.8%)	40 (85.1%)	1.11 (0.41–3.01)	0.839
Vein involvement	54 (67.5%)	34 (72.3%)	1.26 (0.57–2.78)	0.568
Arterial involvement	7 (8.9%)	5 (10.6%)	1.22 (0.37–4.10)	0.761

*p* < 0.05 are in bold

^OR (Chi-squared test) for categorical variables and T (Ordinary least squares regression) for continuous variable; ^&^LODDS = log odds of positive lymph nodes

## Discussion

This study showed that in resected PDAC of the head/neck of the pancreas, Ca 125 outperforms Ca 19.9 as a biomarker. The prognostic value of Ca 125 extends beyond anatomic tumor features, as shown by the prediction of survival in R-PDAC, BR-PDAC, and LA-PDAC. Ca 19.9 predicted OS in R-PDAC, but failed to do so in both BR-PDAC and LA-PDAC. Therefore, Ca 125 should be included among the non-anatomic factors contributing to the new concept of biologic resectability and should be routinely assessed in surgical candidates with PDAC.

Ca 19.9 is currently considered the main tumor marker for PDAC [11–13], but is falsely negative in Lewis negative patients [15], and is falsely positive in patients with biliary obstruction (irrespective of etiology) [16] or cholangitis [17], in patients with pancreatitis [43], and in patients with other cancers of the digestive tract (including periampullary cancers that, sometimes, can show radiological features mimicking PDAC) [44]. Overall, in PDAC, Ca 19.9 shows a median sensitivity of 79% (70–90%) and a mean specificity of 82% (68–91%) in PDAC [45]. Ca 19.9 is not the perfect tumor marker for PDAC.

In this study, lack of prognostic correlation between Ca 19.9 levels and BR-PDAC e LA-PDAC could be explained by the role of Ca 19.9 in the selection of surgical candidates with this local tumor stage. In this respect, selection is particularly stringent for LA-PDAC. Low (and especially decreasing) levels of Ca 19.9 are among the major factors permitting surgery in this subgroup of patients [26]. Once Ca 19.9 levels are low further prognostic implications of this tumor marker may not be evident anymore.

After this point, assay of Ca 125 should be seriously considered. Prognostic implications of Ca 125 levels in PDAC were first proposed over 25 years ago [46], but never gained widespread popularity and acceptance. Levels of Ca 125 are not influenced by the status Lewis antigens as well as the presence of biliary obstruction and cholangitis. A recent study from our group showed that Ca 125 is among the main factors predicting survival in LA-PDAC, when these patients are selected for surgery based also on low Ca 19.9 levels [26]. In the current study, Ca 125 predicted survival irrespective of the category of anatomic resectability. In BR-PDAC and LA-PDAC, survival correlation persisted even after correction for confounding factors.

Considering the cellular mechanism Ca 125/MUC 16 is involved with [19–22], it has been proposed that higher Ca 125 levels could specifically reflect the metastasis-associated tumor burden of PDAC [25, 47]. Ca 125 could, therefore, be used along with Ca 19.9 for prognostic anticipation in PDAC, and could be especially useful in patients with low Ca 19.9 levels following neoadjuvant treatments to further refine the selection of surgical candidates. In patients with LA-PDAC, who require truly extensive surgery with its inherent risk of major surgical complications [34], showing also low Ca 125 levels could among the factors helping to select the best biological candidates. Interestingly enough, the cut-off level that was identified in this study to define patients with high Ca 125 levels in BR-PDAC (19.3 U/mL) is similar to the one (18.6 U/mL) that was used in a previous study to identify the patients who could benefit from resection among those with metastasis in para-aortic lymph nodes [48]. This cut-off is also pretty similar to the one used in an additional study (18.4 U/mL), to identify patients with PDAC expressing a metastasis-associated gene signature causing early metastases and poor survival [47]. In this study, in patients with BR-PDAC and LA-PDAC, high Ca 125 levels were associated with higher T stage, N stage, LN ratio and tumor grading. Considering that tumor size (T stage), lymph nodes status (metastatic ratio), and grade of tumor differentiation are independent prognostic factors for the development of liver metastasis [49], it is clear that high Ca 125 are associated with higher probability of occult distant metastasis resulting in shorter survival perspectives.

This study has several limitations. First, the number of patients was limited, mainly because of stringent inclusion criteria (above all, availability of Ca 125 assay, that is not routine in PDAC). The number of patients could have been increased by expanding the study period, but we preferred to avoid patients treated before 2010 due to lack of effective chemotherapy and after 2018 due to the short period of follow-up. Second, tumor markers were often assayed at different laboratories resulting in (presumably small) variability of results. Third, only few patients undergoing neoadjuvant chemotherapy had Ca 125 assayed at the time of diagnosis, making it impossible to define the prognostic implications of decreasing levels of this tumor marker following oncological treatments. Fifth, data about administration of adjuvant therapies (especially type, and dose) were not available for all patients. However, assuming that administration of adjuvant chemotherapy in the setting of PDAC is standard of care, if permitted by the performance status of the patient, having considered among possible confounders age, ASA class, and occurrence post-operative complications should have reduced the impact of missing details on adjuvant treatments.

In conclusion, we have reported the prognostic implications of Ca 125 across anatomic categories of anatomic resectability in PDAC and we have identified a cut-off level of Ca 125 that correlates with pathologic features predicting earlier tumor recurrence. Therefore, we believe that Ca 125 should be assayed in all patients with PDAC who are possible surgical candidates.

## Data Availability

Data repository is not online available.
